# Dengue-Associated Posterior Reversible Encephalopathy Syndrome, Vietnam

**DOI:** 10.3201/eid2402.171634

**Published:** 2018-02

**Authors:** Nguyen Thi Hoang Mai, Nguyen Hoan Phu, Ho Dang Trung Nghia, Tran My Phuong, Du Trong Duc, Nguyen Van Vinh Chau, Bridget Wills, Choie Cheio Tchoyoson Lim, Guy Thwaites, Cameron Paul Simmons, Sophie Yacoub

**Affiliations:** Hospital for Tropical Diseases, Ho Chi Minh City, Vietnam (N.T.H. Mai, N.H. Phu, H.D.T. Nghia, T.M. Phuong, D.T. Duc, N.V.V. Chau);; Oxford University Clinical Research Unit, Wellcome Trust Major Overseas Programme, Ho Chi Minh City (N.T.H. Mai, N.V.V. Chau, B. Wills, G. Thwaites, C.P. Simmons, S. Yacoub);; University of Oxford, Oxford, UK (B. Wills, G. Thwaites);; National Neuroscience Institute, Singapore (C.C.T. Lim); University of Melbourne, Melbourne, Victoria, Australia (C.P. Simmons);; Imperial College London, Hammersmith Campus, London, UK (S. Yacoub)

**Keywords:** dengue, neurologic, posterior reversible encephalopathy syndrome, PRES, encephalopathy, acute demyelinating encephalomyelitis, ADEM, endothelial dysfunction, meningitis/encephalitis, viruses, Vietnam

## Abstract

Dengue can cause neurologic complications in addition to the more common manifestations of plasma leakage and coagulopathy. Posterior reversible encephalopathy syndrome has rarely been described in dengue, although the pathophysiology of endothelial dysfunction likely underlies both. We describe a case of dengue-associated posterior reversible encephalopathy syndrome and discuss diagnosis and management.

On December 20, 2015, a 55-year-old woman in Vietnam sought medical care at her local hospital near Ho Chi Minh City with 1 day of fever, muscle aches, and anorexia. She had no remarkable medical history; she did not take any medications and had not received any recent vaccinations. Initial hematology and biochemistry tests were normal, but a rapid test for dengue nonstructural protein 1 antigen was positive. She remained hemodynamically stable and did not experience any bleeding or have evidence of plasma leakage. On December 24, she had a generalized convulsion and was transferred to the intensive care unit at the Hospital for Tropical Diseases.

At admission, her Glasgow Coma Scale score was 10/15 (eyes 3, motor 4, voice 3); she was confused, and speech was slurred. Her temperature was 37.5°C, heart rate was 100 beats/min, blood pressure was 140/90 mmHg, and respiratory rate was 18 breaths/min. Cardiorespiratory and abdominal examinations were normal. Neurologic examination demonstrated increased tone in upper and lower limbs and bilateral upgoing plantar reflexes; assessment of power was difficult because of generalized rigidity. Examination of her eye movements demonstrated vertical gaze palsy, but other cranial nerves were intact.

Blood tests showed hemoglobin level of 14 g/dL, hematocrit level of 42.2%, a white cell count of 11 × 10^9^ cells/L, and a platelet count of 100 × 10^9^/L. A test for dengue nonstructural protein 1 antigen remained positive, but PCR was negative. Urea and electrolyte levels were normal, but liver transaminases showed elevated aspartate aminotransferase of 386 U/L and alanine aminotransferase of 242 U/L. Chest radiograph was unremarkable, but a brain computed tomography scan demonstrated bilateral cerebral and cerebellar white matter hypodensities. Lumbar puncture demonstrated an opening pressure of 12 cm H_2_O, a cerebrospinal fluid (CSF) cell count of 7 cells/µL, a red cell count of 4 cells/µL, protein level of 4.4 g/dL, glucose level of 4.76 mmol/L (10.4 mmol/L in blood), and lactate level of 3.25 mmol/L. Dengue virus IgM was detected in CSF; Japanese encephalitis virus IgM was negative. CSF dengue and herpes simplex virus PCR were negative. Microbiologic cultures performed on blood and CSF were sterile.

Magnetic resonance imaging (MRI) of the brain, performed the following day, demonstrated bilateral symmetric high signal on T2-weighted and fluid attenuation inversion recovery images, involving periventricular and deep cerebral white matter (Figure, panel A). The differential diagnoses included encephalitis or acute demyelinating encephalomyelitis (ADEM), and a course of intravenous methylprednisolone was started (1 g/d). After 5 days, this regimen was converted to oral prednisolone (60 mg/d), tapering over 5 days. Phenobarbital was administered for 1 week for seizure control. Repeated neurologic assessment on day 5 revealed normal eye movements and improved rigidity but total left-sided hemiplegia. The patient gradually improved over the next 4 weeks. A repeated MRI 7 weeks later (February 17) demonstrated almost complete resolution, with minimal residual white matter abnormalities (Figure, panel B). The patient was discharged for rehabilitation on February 21.

**Figure:**
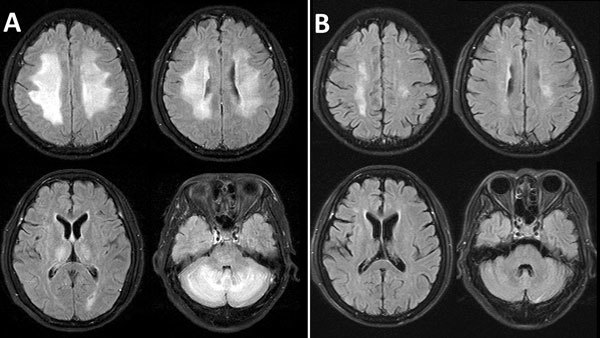
Fluid-attenuated inversion recovery magnetic resonance images of the brain of a 55-year-old woman with dengue-associated posterior reversible encephalopathy syndrome, Ho Chi Minh City, Vietnam. A) Bilateral abnormal nonenhancing, confluent high signal in the periventricular and deep cerebral white matter of the high frontal parietal area and cerebellar hemispheres, thalamus, and pons. B) Almost complete resolution of abnormal findings 7 weeks later, after treatment.

The diagnosis did not fit with dengue encephalitis because of a lack of CSF pleocytosis and the high protein levels; the presence of dengue IgM in the CSF was likely secondary to vascular disruption rather than intrathecal production. Review of the MRIs, which demonstrated early reversible white matter changes rather than delayed multifocal discrete lesions associated with ADEM, were diagnostic of dengue-associated posterior encephalopathy syndrome (PRES) (*1*).

PRES is an acute neurologic syndrome, typically in patients with blood pressure fluctuations or metabolic derangement (*1*). However, PRES has been recognized to complicate various infections accompanied by normal blood pressure (*2*,*3*). Characteristic radiographic findings include bilateral white matter changes in areas supplied by the posterior circulation but can be diffuse, as described in this case, and resolve over weeks. High CSF protein levels correlate with cerebral edema and disease severity (*4*). The pathophysiology of PRES is thought to involve disruption to cerebral blood flow autoregulation, endothelial dysfunction, and vasogenic edema (*1*).

Most dengue infections cause a self-limiting febrile illness; however, life-threatening complications can occur, including increased capillary permeability, causing plasma leakage and shock. Like PRES, endothelial dysfunction is thought to underlie the capillary leak (*5*). Severe dengue can also occur with specific organ involvement (including neurologic) and without other severe features, as defined by the 2009 World Health Organization classification (*6*). Various neurologic manifestations have been described in dengue; however, PRES has been suspected in only 2 other reported cases (*7**,**8*), possibly because of underreporting or misdiagnosis, especially given the limited access to neuroimaging services in dengue-endemic areas and the common assumption that PRES diagnosis requires hypertension or metabolic derangement to be present. Unlike ADEM, PRES usually only requires supportive treatment. 

This case highlights the need to consider PRES in dengue patients with neurologic symptoms and that PRES should be distinguished from encephalitis or ADEM. The high CSF protein levels and characteristic MRI findings we have described could assist clinicians in dengue-endemic areas.
